# Factors influencing compliance in RRD patients with the face-down position via grounded theory approach

**DOI:** 10.1038/s41598-022-24121-9

**Published:** 2022-11-25

**Authors:** Yahong Li, Jining Li, Ying Shao, Ronghua Feng, Jinkun Li, Yajian Duan

**Affiliations:** 1 Shanxi Bethune Hospital, Shanxi Academy of Medical Sciences, Tongji Shanxi Hospital, Third Hospital of Shanxi Medical University, Taiyuan, 030032 China; 2 Tongji Hospital, Tongji Medical College, Huazhong University of Science and Technology, Wuhan, 430030 China; 3 Department of Health and Environmental Sciences, Xi’an Jiaotong-Liverpool University, Suzhou, 215123 China; 4 Institute of Population Health, University of Liverpool, Liverpool, L69 3BX UK; 5 Shanxi University of Finance and Economics, Taiyuan, 030006 China

**Keywords:** Human behaviour, Health care, Health occupations

## Abstract

Patients with rhegmatogenous retinal detachment (RRD) require face-down positioning (FDP) for 3–6 months or longer after pars plana vitrectomy (PPV) combined with silicone oil (SO) tamponade. This paper aimed to identify the factors that influenced FDP compliance. This study adopted semi-structured interviews with patients who require FDP after SO tamponade. Constructivist grounded theory was utilized in this study. The qualitative data was analyzed and coded via NVivo 11.0 through open coding, axial coding and selective coding. Twenty-four RRD patients were involved. The interviews yielded five main themes that defined home FDP compliance were identified: posture discomfort, doctor-patient communication, psychological factors, occupational character, and family factors. A theoretical model of the influencing factors of postural compliance of FDP was constructed based on the interview analysis. A variety of factors can affect FDP conformity. We can increase compliance of RRD patients by enhancing comfort, encouraging doctor-patient communication, providing comprehensive care, promoting community-based intervention, and strengthening family education.

## Introduction

**Figure 1:**
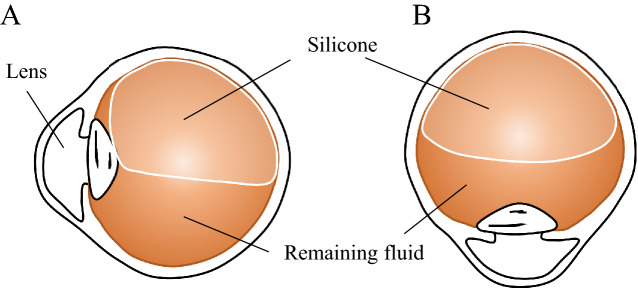
A diagrammatic representation of the silicon tamponade position in two different head postures. (**A**) shows silicon filled eye during face down position. (**B**) shows silicon filled eye during forward-facing position.

**Figure 2:**
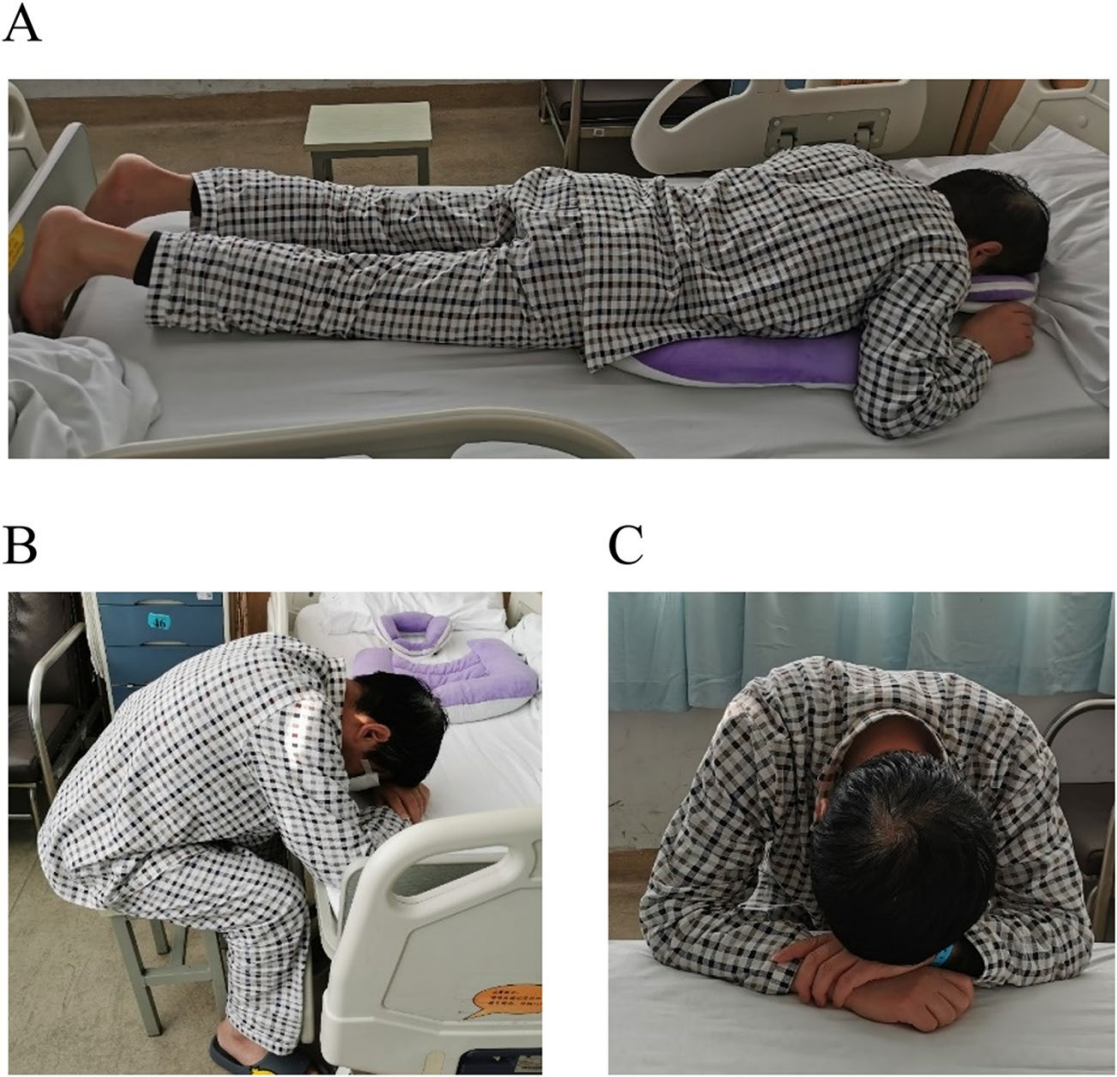
Patient’s face down position. (**A**) shows the patient’s prone position. (**B**,**C**) shows the front and side of the patient’s sitting posture.

Rhegmatogenous retinal detachment (RRD) is a potentially blinding condition characterized by the separation of the retinal neurosensory layer from the pigment epithelial layer^1^. The difficulty of treating RRD varies depending on the location, size, and duration of detachment and the age of the patients^2^. If surgery is not performed in time, it can lead to permanent loss of vision^3^. Retinal detachment is a heterogeneous condition, ranging from limited chronic detachments to high bullous detachments, which can progress slowly or rapidly with subsequent vision loss. The difficulty coefficients of surgery are significantly increased, from a single localized retinal tear to complex cases of advanced vitreoretinopathy^4^. Retinal detachment mainly occurs in patients with older age, high myopia (myopia degree greater than -6.00D), history of ocular trauma, or a family history of retinal detachment^5^. Pars plana vitrectomy (PPV) is a common surgical procedure for retinal detachment^6,7^. After vitrectomy, SO is often filled in the vitreous cavity, and the retina is pressed by its buoyancy to maintain the pressure in the vitreous cavity (Fig. 1), which can increase the reattachment rate and anatomical success rate of the retina^8^. 3–6 months after SO tamponade, patients need to maintain a face-down positioning (FDP) every day. Regardless of the body position, such as prone, seated and standing, the eyes must remain parallel to the floor^9^ (Fig. 2). Patients was recommended to keep this position even walking^10^. And they can change to another position actively as desired^11^. After discharge, the patients need to adhere to the FDP according to the doctor’s order, regularly go to the ophthalmology for reexamination, and adjust the FDP time according to the retinal condition until the SO is removed.

Previous studies have been based primarily on patient compliance with in-hospital FDP after SO tamponade. Studies have shown that patients’ compliance with the FDP in hospitals gradually decreased over time^12^. Clinical studies have found that compliance with home FDP may be even worse^13^. Poor compliance may result in a longer SO tamponade time, causing a series of complications like high intraocular pressure, cataract, SO emulsification, keratopathy, and the formation of the epiretinal membrane^14,15^, as well as an extended retinal reattachment time or unsatisfactory vision improvement, which may trigger doctor-patient contradictions and disputes. The following questions deserve further consideration. What are the challenges that patients face when they are in FDP? What features of medical personnel should assist patients in improving their posture?

Previous studies focused on the degree of standardization of FDP in hospital settings, which cannot be used for physician assessment and clinical guidance of patients' long-term home FDP. In addition, previous literatures were quantitative studies and overlooked the effects of factors such as self-perception, beliefs, real-life stress and family support on FDP adherence. In conclusion, there is a lack of a comprehensive study on the influencing factors that affect patients’ FDP compliance, especially home adherence. In this paper, we use a qualitative research method to investigate the compliance of long-term home FDP in patients with RRD after hospital discharge, taking into account psychological and family social factors, in order to provide a theoretical basis for improving patients’ compliance with long-term FDP after hospital discharge.

Qualitative research is a method in which the researcher analyzes, categorizes, and refines certain common characteristics and connotations, based on subjective information about the research subject obtained from in-depth interviews, participant observation, inquiries into archives or records, and articulates the findings in writing^16,17^. Grounded theory is a qualitative research method that employs systematic procedures to create and infer a theory based on real-world facts about a certain phenomenon^18^. Constructivist ground theory developed by Charmaz, assumed that the theory is developed by researcher based on data, constructed with participants usually by interviews^19^. The unique characteristics of constructivist ground theory, simultaneous data collection and analyses, theoretical sampling, constant comparison at each stage of analysis, development of memos for reflexive and comparative analysis, are used to guide the analytical process of building a theory^20^. This paper intends to conduct a qualitative study using constructivist grounded theory to investigate the factors affecting FDP compliance in RRD patients who residence at home after SO tamponade, with the goal of identifying positive coping styles and intervention measures to improve FDP self-management in these patients.

## Methods

This qualitative study is based on individual semi-structured interviews guided by grounded theory. Grounded theory creates a theoretical model “through observing a study population and developing a comparative analysis of their speech and behavior”^16^. This approach helps to reveal the factors that influence home FDP adherence, as well as to better understand and grasp the reasons for poor compliance in patients with RRD.

### Constructivist grounded theory

The guideline of using constructivist grounded theory have some common characteristics, including theoretical sampling, constant data collection, analysis, and comparison; as well as, memo writing and theoretical development^20^. Theoretical sampling is applied to explore an unclear analysis category, which contributes to define the specific participants. After the semi-structure interview outline is created, the researchers will start simultaneous data collection and analysis. Constant comparison is used to analyses and build categories by comparing data with data, codes with codes, and incidents with incidents^21^. Memoranda are an informal approach to record researchers’ idea, reflection, and possible connection before starting and when collecting and analysis data^22^.

### Data collection

To ensure the diversity of the research team and the rigor of the interview content, this study included a total of 12 experts, three each of ophthalmologists with senior professional titles, attending psychiatrist, nursing experts with senior professional titles, and psychologists with senior professional titles.

The participants were recruited from the ophthalmic ward of a general hospital in Taiyuan, China. This study focused on RRD patients who require FDP after vitrectomy with SO tamponade. The patients invited to the study had the following characteristics: (1) diagnosed with RRD, (2) PPV surgery with SO tamponade, (3) FDP for at least 3 months after surgery, and (4) all patients signed an informed consent form and voluntarily agreed to participate in the study. Exclusion criteria: (1) communication barriers, including psychological problems, (2) unable to complete the interview and communication independently, and (3) refuse to accept telephone interview.

Theoretical sampling was applied in this study^23,24^, which was suggested by Glaser and Strauss (1967) as “the process of data collection for generating theory whereby the analyst jointly collects, codes, and analyzes his data and decides what data to collect next and where to find them, in order to develop the theory as it emerges” (p. 45). Data were collected through one-on-one telephone semi-structured interviews from November 2021 to April 2022. The interview length of each patient was around 30 min, and the interviews were conducted in a quiet or private environment. The researchers consist of twelve personnel with senior professional titles in different occupations. The outline for the semi-structured interviews was developed by researchers after reviewing similar papers and group discussions, and the initially developed outline was adjusted after the pre-interview. The researchers evaluated the FDP of RRD patients and conducted in-depth interviews with the patients with poor self-management. The interviewer asked, “Is your self-management of FDP consistent with the requirements of medical staff?”. The final questions were as follows: (1) “Could you tell me your feelings and thoughts in the FDP?”; (2) “What are the main difficulties in maintaining this position?”; (3) “How do you relieve when you are feeling uncomfortable?”; (4) “How does FDP make changes to your daily life?”; (5) “Would you share any other information not mentioned?” (Supplementary table 1). On the premise of obtaining the informed consent of the patient, the interview content was recorded in the form of audio recording. During the interview, the patients were encouraged to explain freely without comment, guidance, or tendency induction, the researchers listened carefully and audio recorded. The researcher determined whether the information saturation was reached, according to whether the repeated information was heard, whether there were new influencing factors in the data analysis, and whether the selected interviewees were comprehensive. The interview ended when the researcher thought there was no new information in the interview and the narrative data was saturated. The data was saturated or no newer information showed up until 24 cases, hence the interview was finished with 24 participants. Memos were written by interviewers to record the key words or sentences, titled by virtue of the category or property, for the purposes of future sorting and integrating^22^.

### Data analysis

The interview data were analyzed based on grounded theory, which is characterized by a constant comparative method of analysis (Glaser & Strauss, 1967). In this study, the process of analysis began at the same time as data collection, as well as, constant comparison^25,26^. The primary researcher programmed three-level coding of the proofread records using the qualitative software NVivo 11.0 (https://www.qsrinternational.com/nvivo-qualitative-data-analysis-software/home) to identify specific topics^27^. (1) Open coding. The transcribed text is treated with privacy, and the document name is set to be replaced by the number when the respondent is included, such as P1, P2, …, P24. Define the content of each paragraph, find out and encode the subtopics. (2) Axial coding. Classify the relationship and logical order obtained by open coding, refine the theme, and integrate and conceptualize the data. (3) Selective coding. It refers to the selection of a “core category” that plays a leading role after systematic analysis in the category. Memo writing was also applied to data analysis to get the conceptual and theoretical ideas through free writing by the analysts^22^. Researcher reflexivity was the important part of the coding process. The memos, field notes, and the feeling of the participants combined to add credibility to the findings.

### Ethical consideration

Research ethics approval was obtained from Shanxi Bethune Hospital (YXLL-2022-090). The research was performed in accordance with relevant guidelines, and informed consent was obtained from all participants or their families. It has been performed in accordance with the Declaration of Helsinki. The dataset analyzed during the current study are not publicly available but are available from the corresponding author on reasonable request.

All of the interview participants had the cognitive ability to read and understand the participant’s information sheet and agreed to participate in the study voluntarily by signing the informed consent sheet. Before the interview, all researchers had no prior relationship with the participants. The researchers introduced themselves and explained the purpose and methods of the study. Participants can drop out of the study at any time. The researchers promised not to use any personal identifiers in research reports or publications.

### Ethics approval

This study involves human participants and was approved by the Shanxi Bethune Hospital Ethics Committee.

## Results

**Figure 3:**
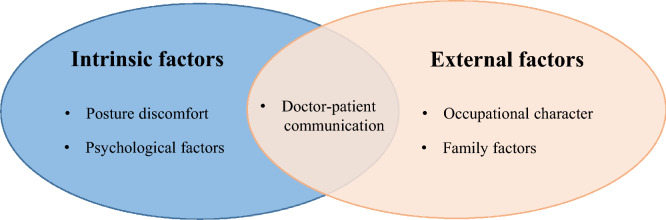
Schematic diagram of the theme of influencing factors of FDP postural compliance in patients with retinal detachment. According to grounded theory, five themes were identified. Among them, posture discomfort and psychological factors are the patient’s intrinsic factors; occupational character and family factors are the extrinsic factors; and doctor-patient communication is the overlapping factor.

A total of 24 patients were included in this study. The median age was 59 years old (age range, 28 ~ 75 years) (Table 1). The demographics information also included marital status (married, divorced), working status (working full-time, not working, and self-employed), medical insurance, etc. The interview data was analyzed by applying three-level coding, including open coding, axial coding, and selective coding, and conceptualized and integrated into the core category to seek the internal relationship in the data. Finally, five themes and twelve subthemes were extracted from the influencing factors of postoperative home FDP compliance of RRD patients with SO tamponade (Fig. 3) (Table 2).

**Table 1:** Demographic details of study participants (*N* = 24).

Subject characteristics	N	%
**Gender**		
Male	16	66.67
Female	8	33.33
**Age in years**		
< 60	14	58.33
≥ 60	10	41.67
**Education level**		
Primary school	4	16.67
Middle school	11	45.83
Undergraduate	9	37.50
**Place of residence**		
In Taiyuan city	7	29.17
Outside Taiyuan city	17	70.83
**Marital status**		
Married	21	87.50
Divorced	3	12.50
**Work status**		
Working full-time	6	25.00
Not working	16	66.67
Self-employed	2	8.33
**Health care**		
Yes	22	91.67
No	2	8.33
**Extend of retinal detachment**		
1 quadrant	20	83.33
2 quadrants	2	8.33
3 quadrants	1	4.17
4 quadrants	1	4.17
**Complications**		
No	8	33.33
Yes	16	66.67

**Table 2:** Identified themes and subthemes.

Themes	Subthemes
Posture discomfort	Physical discomfort
	Eye discomfort
Doctor-patient communication	Healthcare worker factors
	Patient factors
Psychological factors	Slackening of thinking
	Low self-efficacy
Occupational character	Character of work
	Balancing revenue and expenses
Family factors	Family burden
	Family intervention

### Posture discomfort

There was no doubt that physical and eye discomfort caused by long-term FDP was an essential factor for the poor compliance of RRD patients. Although the doctors and nurses informed the patients of the possible discomfort of the eye and the body of long-term FDP before the operation, the patient still felt unable to maintain this position for 3 months or even longer.

#### Physical discomfort

Through the word frequency query function of QRS NVivo 11.0 software (minimum length set to “2”). It was found that “no”, “comfortable”, “feel”, and “now” were the most frequent word throughout the interview, suggesting that posture discomfort was the main factor affecting patients’ self-management. Most patients chose the prone or seated position for FDP. In the prone position, a U-shaped pillow was placed under the face, but prolonged time in this position can lead to poor breathing and physical discomfort. In the seated position, the U-shaped pillow was usually not placed, and breathing was maintained by placing the hand under the forehead, which may lead to hand discomfort (Fig. 2). A small number of patients can only choose to sit in the FDP due to reasons such as obese persons, the elderly, or the patients with the installation of a heart stent.

“I’m too skinny to hold the FDP position because when I get down, the bones in my chest pinch and I can’t breathe well. My son bought a metal frame for me to sit on and get down on.” P3

In addition to chest discomfort, other patients reported discomfort in other parts of the body as well.

“I was sitting in the FDP and my waist was mainly uncomfortable.” P1

“My head was uncomfortable when I got down. Sometimes I felt nauseous and wanted to vomit.” P5

“That position made it difficult for me to breathe, and my throat, arms and shoulders were uncomfortable.” P13

“Whole body was uncomfortable” P23

#### Eye discomfort

In addition to physical discomfort, some patients experienced eye swelling after SO tamponade, which may increase discomfort and decrease compliance.

“My eyes were swollen. The intraocular pressure (IOP) rose when I didn’t lower my head in the early stage.” P10

“When I lied down, my eyes were swollen and blurred.” P22

SO may also lead to blurred vision, binocular anisometropia, glare, and a variety of other symptoms, all of which can have an impact on the patient’s daily work and life. Compared with glare, binocular anisometropia had a higher impact on patients. The former can be alleviated by wearing sunglasses or going out in dim light; however, the latter was difficult to overcome. Furthermore, the focus deviation of SO-tamponade patients' eyes might cause inconvenience in daily tasks, taking objects, parking, etc. The reduction of balance caused by focus deviation was linked to some life restrictions on the patients. Patients’ feelings of powerlessness over “simple things” may lead to anxiety in the long-term phase, which had a certain relationship with patients' compliance.

“For example, sometimes things are held in the wrong place and not focused on one point.” P10

“I had no idea how far it (vehicle and wall or roadside) was more than a meter away. There was no sense of distance.” P10

“I was afraid to leave the house. I can’t do anything. I still put in a lot of effort on my own walkway straightly, and I was unable to work.” P23

### Doctor-patient communication

Doctor-patient communication refers to the communication between doctors and patients during the diagnosis and treatment of diseases in order to facilitate the treatment of patients’ diseases and meet their health needs. It is a two-way conversation. Compliance with the FDP must be closely monitored by the medical staff and the patient (or the patient’s family). Lack of clarity in medical staff advices or lack of proactive questioning about FDP adjustments, adjustment made by patient, and less follow-up visits may reduce FDP compliance and increase some complications.

#### Healthcare worker factors

From a health care perspective, inadequate patient education on FDP, unclear recommendations for FDP time modifications, and infrequent follow-up visits to patients are the main reasons for poor FDP compliance. The low knowledge and acceptance reflect the lack of patient education by healthcare providers. Lack of clarity in medical advice occurred mainly during the discharge and the follow-up process. Specifically, the FDP time and posture requirements may not be clearly explained at the time of discharge; and at the follow-up time, the doctor may forget to explain the FDP time adjustment. This often leads to a misunderstanding between the doctor and patient. The doctor may think that no special instructions mean the medical advice is the same as before; the patient may think there is no special emphasis therefore may reduce the time of FDP or even not to maintain it.

“They (doctors and nurses) did not explain the length of time and importance of FDP. They just said I had to get down.” P2

“All this time, the doctor probably thought I might not be so severe, and at first she said it was necessary to get down for five or six hours a day, and then she did not say (change) and I did not ask the doctor. I just did not keep lying down.” P20

The low number of follow-up visits is an inevitable reality. Medical staff are stressed and busy, and a great deal of time and energy is devoted to inpatients and outpatients, which makes extended care a relatively inadequate. In addition, some patients are far from the hospital and do not feel the care and encouragement of the medical staff, which also makes them less compliant.

“When I was in the hospital, the nurses checked the accuracy and length of postoperative FDP several times a day, gave guidance and advice, encouraged me, and told me the posture was important. When I went home, there was no one to monitor and remind me or care if I had enough time to do it.” P15

#### Patient factors

Regarding the patients, there are three main reasons for poor adherence. First, patients may forget to ask about FDP adjustments; in addition, they become tired of adhering to the position during follow-up and are likely to make their own adjustments, mainly by reducing the duration of the FDP. Second, they may have reduced their time in the FDP on their own when they knew their eye condition had improved and the doctor did not “emphasis” the FDP time. Third, some currently employed patients prefer to come in on weekends for follow-up visits; however, the outpatient time for doctors is usually scheduled on weekdays. For these three reasons, they may modify and adjust the FDP time on their own. Fewer follow-up visits also lead to poor adherence

“I did not go for a follow-up last time and did not ask my doctor how to get down, and I lied in a prone position less now.” P16

“I need to work on weekdays and have time on weekends. However, the doctor I wanted to make an appointment with was not available on the weekend and I did not make an appointment with another doctor for a follow-up appointment.” P9

### Psychological factors

The influence of psychological factors on FDP is mainly in two aspects. First, FDP needs to be adhered to for a long time. As time passes, some postoperative discomfort relieves and mood relaxes gradually, and patients may become noncompliant and seek a more comfortable body position. Second, the lack of improvement in vision during long-term adherence to FDP may lead to a lack of self-efficacy. Chronic blurred vision raises doubt about the recovery of vision after SO extraction.

#### Slackening of thinking

As time passes and some symptoms subside, patients may become less compliant. Some patients believe it is human nature to seek a more comfortable body position.

“When I first started, walking upright for a little longer made my eyes uncomfortable, and now that I can walk without any discomfort, I don’t maintain the required prone position as strictly as I did when I first had the surgery.” P10

“I felt that I have made a full recovery after 3 months. I was so tired to lie in the prone position, I stopped doing it.” P23

“They (eyes) kept level with the ground when I was in the hospital. But when I returned, I always felt as if it had been a waste of time.” “I figured it didn’t matter whether I lay down or not.” P13

#### Low self-efficacy

Patients’ compliance behaviors were directly influenced by self-efficacy. Although medical staff indicated that maintaining the FDP did not help the patient’s vision during SO tamponade, however, as time passes, patients may worry about whether their vision will improve after the SO is removed.

“After 3 months of staying prone position, my vision did not improve, so I spent less time on that.” P25

### Occupational character

Each patient has different division of labor. Inevitably, different positions correspond to different levels of salary. This makes the economic level of each family different and has a great impact on the standard of living.

#### Character of work

The analysis of the interview results suggests that the compliance of currently employed patients may be relatively better than that of unemployed patients. This is mainly related to the fact that the unemployed are mostly composed of a group of farmers who perform more physically demanding work. Secondly, the unemployed and freelancers have lower health insurance reimbursement rates than the employed or retired.

“We live so far away that it costs 500 or 600 RMB for the travel. I am a farmer and I am over 80 years old. I did not have much source of income. The cost of the surgery was very high for me and health insurance only reimbursed half of it.” P3

#### Balancing revenue and expenses

For some patients, the cost of surgery accounts for a large portion of household expenses. In addition, these patients are unable to perform heavy work for up to 6 months after surgery, and their family income is significantly reduced. The above reasons lead to an imbalance of income and expenditure for these patients’ families, forcing them to work and thus not securing enough FDP time.

“I have been maintaining FDP for 6 months, and I have spent tens of thousands of RMB on this surgery, and I have no source of income, so I definitely have to find something to do, otherwise I can’t afford to support my family.” P23

### Family factors

Family factors inevitably influence patients’ compliance with FDP. In the Chinese culture, each family member feels obligated to perform household chores, especially women. Generally, family members can accept that patients do not do housework in the short term due to the maintenance of the FDP after surgery; however, in the long term, most female patients cannot avoid doing housework.

#### Family burden

Some female patients need to take care of their families and do housework after surgery. The burden of housework poses a challenge to their FDP time. Some patients felt they had spent a lot of money on surgery, and not doing housework added to their feelings of guilt.

“It was winter, and I still needed to cook and pick up the kids. When I had free time, I just lied down for a while.” P23

#### Family intervention

The reasons for poor FDP compliance were related to the lack of knowledge of FDP management among family members. Some family members did not understand the necessity and importance for FDP, believed that staying in bed all the time was not good for the patient’s health, and even urged them to reduce the time of FDP.

“After 100 days, I did not get down anymore, and my son kept scolding me, saying that it must be bad for my health if I lied there every day and did not move back and forth.” P3

## Discussion

Grounded theory is a theoretical model “through observing a study population and developing a comparative analysis of their speech and behavior”^16^. Constructivism as the philosophical foundation of ground theory. Constructivist ground theory assumes that as human being, the researcher is biased, since they have their own opinions, values, and professional cognition. In constructivist ground theory, researchers assume that data and analyses are social constructions, and participants and researchers alike construct meanings. Therefore, any analysis developed through research can both reflect the participants’ experiences and the researcher’s perception of the phenomenon. Evidently, the constructivist ground theory, differs from the original version of ground theory by Glaser and Strauss, which claimed that the original theory is discovered in the data, and it is not influenced by the researchers. Constructivist ground theory can be considered as an innovation of ground theory^20^.

Under the guidance of constructivist grounded theory, this study carried out a qualitative study in the form of in-depth interviews, and obtained five themes of home FDP compliance of SO filled RRD patients, namely posture discomfort, doctor-patient communication, psychological factors, occupational character, and family factors (Fig. 3). On this basis, the concept of core categories and their significance were further refined, and a theoretical model of factors influencing of FDP compliance in SO tamponade patients was constructed. The model profoundly reflects the patient's feelings (posture discomfort) and psychological factors, which are intrinsic factors. Occupational character and family factors are extrinsic factors. Doctor-patient communication is included within both. The four factors were not considered in the clinical quantitative analysis, except for physical and eye discomfort. This study provides the corresponding theoretical basis and data support for improving the compliance of FDP in patients with SO tamponade after PPV. According to the factors influencing compliance, medical staff should improve posture comfort and family-centered educational interventions, which are effective ways to help patients.

Previous studies have shown a number of ways to improve compliance with FDP and to make its application more pleasant for patients. Postural comfort after SO tamponade is strongly associated with FDP compliance^28^. Most of the investigated patients used ordinary U-shaped pillows to maintain their posture, with poor air permeability. The hand long-term support on the forehead resulted in neck, arm or lumbar discomfort. Providing comfortable, inexpensive and easy-to-use support for patients is essential to improve compliance with FDP^29^. Upper body support from the chest to the pelvis helps to place the upper body above the lower body, thereby reducing back stress. Limb exercises and postural alternations during FDP maintenance are also useful in the treatment of back, neck, shoulder and lower back discomfort^28^. In addition, the study has shown that the simple oil massage can reduce immediate pain in patients undergoing FDP, while aromatherapy massage with essential oil had a long-term effect on pain relief^30^. Patients are advised to choose a physiotherapy bed. The facial sinkhole in front of the bed is designed to accommodate the patient’s facial breathing. To divert attention, increase comfort and pleasure, and make FDP maintenance more user-friendly, patients can also see electronic devices or drink water through the sinkhole. Hara et al.^11^ found that patients who used the TV-watching table was more satisfied than the patients who did not use it. Therefore, the improvement of care system is crucial to physical and mental health of patients, especially for the patients with elderly, obesity, coronary heart disease, and vertebral column troubles^31,32^. Due to the high refractive index of SO, the refractive index of the eye changes significantly and the visual acuity decreases significantly^10^. For patients with postoperative refractive partials, glasses are recommended for partial correction to facilitate daily walking and living.

By standardizing medical practices, ambiguity in medical prescriptions due to omissions can be greatly reduced. We need to develop a review process for SO-filled patients. The FDP adjustment protocol, including: PPV procedure time, follow-up time, tear location, and prone time, should be pasted in the medical follow-up records of patients after SO filling to ensure the process is completed. Patients with high IOP need to be reminded to follow up on time, and seek medical attention for special conditions at any time. Doctors can extend the FDP time appropriately, and prescribe IOP-lowering medications according to the patient's condition. Other studies have found that IOP in FDP varies depending on the face-down position; FDP has the lowest IOP in the seated position and the highest IOP in the prone position^9^. In conclusion, encouraging patients to take additional medications and to perform interchangeable FDP may be effective approach to maintain this position. To address the issue of psychological relaxation, follow-up, care, and inquiring patients should be enhanced, and patients should be asked to follow medical advice to reduce complications.

For unemployed patients who are labor-intensive due to their occupational characteristics, a diagnostic proposal can be issued and the patient’s community can be contacted to seek help and solutions to find suitable jobs and alleviate economic stress during the FDP. In addition, hospitals and communities can actively contact relevant social institutions such as women's federations and disability federations to provide assistance to patients with difficult family conditions. Health education is crucial to improve patients' self-care and self-efficacy. To enhance patients’ understanding of primary disease and FDP and to master and standardize FDP, the department can regularly push demonstration action videos in WeChat groups, hold lectures on the Internet to enhance patients’ confidence with examples of recovered patients, and conduct patient education through cases of disease complications and recurrence caused by poor FDP compliance.

The patient’s family plays an important role in the management of FDP. When family members are knowledgeable about self-management, they can act as supervisors and guides to promote higher compliance. Studies have shown that family-centered education greatly improves treatment adherence^33^. Encourage patients' families to actively participate with patients in patient education, learning, and health guidance to increase family attention to patients’ FDP. Attempts to reduce the stress of household chores and prevent patients from compromising their postural time due to family obligations are also effective. At the same time, medical staff and patients’ families should spend more time in accompanying, explaining, and reassuring patients and providing timely psychological support. In conclusion, timely follow-up visits and health education are necessary to improve patient self-management, maximize the standardization of FDP, save medical resources, improve patient satisfaction, and promote doctor-patient communication.

This study has the following limitations: (1) The coding and theoretical model may be subjective. The representativeness of the small sample and the generalizability of the findings have not been tested with a large sample. Whether the theoretical model can explain the influencing factors of FDP compliance still needs to be further explored. (2) The respondents of this study were ophthalmology patients from Bethune Hospital in Shanxi. Considering the differences in economic levels, medical models and health education approaches in different regions, the study results may have some limitations. (3) At present, there is no clear standard for patient's compliance with the FDP. This study may have some bias in determining the compliance of patients according to the consistency between the patient’s position time and the doctor’s requirements, also the standardization of the posture. (4) In order to minimize bias and power asymmetry among different interviewers, a trained physician was selected as the interviewer. This interviewer had a senior professional title in ophthalmology, and was able to give professional explanations about the patient’s condition and postoperative complications or discomfort. However, we must admit that this may not be entirely avoidable .

## Supplementary Information

Supplementary Table 1.

## Data Availability

The datasets used and analysed during the current study available from the corresponding author on reasonable request.
