# Supplementary material for: Factors influencing compliance in RRD patients with the face-down position via grounded theory approach
Source: Sci Rep. 2022 Nov 25;12:20320. doi: 10.1038/s41598-022-24121-9 (PMC9700789; doi:10.1038/s41598-022-24121-9)
Supplement: Supplementary file 1 — Supplementary Table 1. [file 41598_2022_24121_MOESM1_ESM.docx]

Supplementary table Interview Outline of compliance in RRD patients with the face-down position

| **Number** | **Concept** |
| --- | --- |
| 1 | Is your self-management of FDP consistent with the requirements of medical staff? |
| 2 | Could you tell me your feelings and thoughts in the FDP? |
| 3 | What are the main difficulties in maintaining this position? |
| 4 | How do you relieve when you are feeling uncomfortable? |
| 5 | How does FDP make changes to your daily life? |
| 6 | Would you share any other information not mentioned? |
